# Postoperative inflammatory markers are not associated with hidden blood loss after knee arthroscopy

**DOI:** 10.3389/fmed.2026.1783296

**Published:** 2026-03-17

**Authors:** Sheng Li, Wei Wang, A. Liang

**Affiliations:** 1 Department of Orthopaedics, Central Hospital Affiliated to Shenyang Medical College, Shenyang, Liaoning, China; 2 Shenyang Hand Foot Clinical Research Center, Shenyang, Liaoning, China; 3 Department of Basic Medical Sciences, Hainan, Vocational University of Science and Technology, Haikou, Hainan Province, China

**Keywords:** C-reactive protein, hidden blood loss, inflammatory markers, knee arthroscopy, postoperative rehabilitation, white blood cells

## Abstract

**Objective:**

Hidden blood loss (HBL) and systemic inflammatory response are common after knee arthroscopy, but whether they are interrelated remains unclear. This study aimed to investigate if changes in inflammatory markers (∆CRP and ∆WBC) are associated with HBL, independent of known hematological predictors.

**Methods:**

A total of 34 patients undergoing knee arthroscopy were included. Demographic, surgical, and laboratory data were collected. HBL was calculated using a standard formula. Inflammatory markers (CRP and WBC) were measured preoperatively and postoperatively. Linear regression was used to assess the association between HBL and ∆CRP/∆WBC, adjusting for covariates.

**Results:**

A total of 34 patients were included. Postoperatively, the median CRP was 5.36 mg/L (IQR 2.80–12.20), the mean WBC was 9.25 ± 2.07 × 10^9^/L, and the median HBL was 393.60 mL (IQR 273.42–672.97). Postoperative CRP and WBC levels increased significantly compared to preoperative values (*p* < 0.001). However, neither univariate nor multivariate analysis showed a significant association between ∆CRP or ∆WBC and HBL (*p* > 0.05).

**Conclusion:**

Although inflammatory markers increased significantly after knee arthroscopy, they were not associated with HBL. This suggests that postoperative inflammation and hidden blood loss represent distinct pathophysiological processes. Clinicians should understand that while postoperative fever and elevated inflammatory markers are expected, they do not indicate increased blood loss, and HBL itself is not a risk factor for infection.

## Introduction

Knee arthroscopy is one of the most common orthopedic procedures worldwide, performed for a wide range of indications including meniscal tears, ligament injuries, synovial pathologies, and degenerative conditions (1). Despite its minimally invasive nature, patients frequently experience two important postoperative phenomena: hidden blood loss (HBL) and systemic inflammatory response. Hidden blood loss, first described by Sehat et al. (2), refers to blood loss that is not visible intraoperatively or in postoperative drains, accumulating instead in joint spaces and tissue compartments. Studies across various orthopedic procedures have demonstrated that HBL can be substantial—ranging from 287 mL in percutaneous vertebroplasty (3) to over 750 mL in shoulder arthroscopy (4)—and may contribute to postoperative anemia, delayed rehabilitation, and increased length of hospital stay (5). Concurrently, the postoperative inflammatory response, characterized by elevated C-reactive protein (CRP) and white blood cell (WBC) counts, is a physiological reaction to surgical trauma (6). However, in clinical practice, distinguishing between expected postoperative inflammation and early signs of infection can be challenging (7). This distinction is particularly important in arthroscopic surgery, where delayed diagnosis of infection can have devastating consequences for joint function (8). While previous research has identified factors associated with HBL, including hematocrit levels and estimated blood volume (9), the relationship between HBL and the inflammatory response remains poorly understood. Several potential mechanisms suggest a possible connection: hemolysis from tourniquet use may release damage-associated molecular patterns that trigger inflammation (10); oxidative stress from ischemia–reperfusion injury can activate both hemolytic and inflammatory pathways (11); and accumulated blood in the joint space may provoke a local inflammatory reaction (12). Understanding whether HBL and inflammatory markers are related has important clinical implications. If HBL drives inflammation, then strategies to minimize HBL might also reduce postoperative fever and infection risk. Conversely, if they are independent processes, elevated inflammatory markers should not prompt concern about excessive bleeding, and vice versa. Therefore, this study aimed to investigate whether changes in bacterial inflammatory markers (CRP and WBC) after knee arthroscopy are associated with HBL, while accounting for known hematological predictors. We hypothesized that HBL and inflammatory markers would not be significantly associated, reflecting distinct underlying mechanisms.

## Methods

### Ethical approval

The study protocol was approved by the Institutional Review Committee (IRB) of the Central Hospital Affiliated to Shenyang Medical College and followed the tenets of the Declaration of Helsinki. This study obtained informed consent from all participants.

### Study population

We retrospectively reviewed medical records of patients who underwent knee arthroscopy at our institution between January 2024 and December 2024. Inclusion criteria were: (1) age ≥ 18 years; (2) primary knee arthroscopy for any indication; (3) complete preoperative and postoperative (within 48 h) laboratory data including complete blood count and CRP; and (4) availability of complete surgical and anesthesia records. Exclusion criteria were: (1) known coagulation disorders or bleeding diathesis; (2) preoperative anticoagulant or antiplatelet therapy (except low-dose aspirin for cardiovascular prophylaxis); (3) revision arthroscopy; (4) concomitant open surgery; (5) preoperative infection or inflammatory condition; (6) recent corticosteroid injection (< 4 weeks); and (7) incomplete medical records. The specific arthroscopic procedures performed included: diagnostic arthroscopy (*n* = 34), partial meniscectomy (*n* = 28), synovial debridement (*n* = 15), loose body removal (*n* = 9), microfracture for chondral defects (*n* = 6), and anterior cruciate ligament reconstruction (*n* = 4). Multiple procedures were performed in most patients. All surgeries were performed by two experienced orthopedic surgeons using standardized techniques with tourniquet control (250–300 mmHg) and continuous irrigation with normal saline at 50–70 mmHg pressure.

### Injury mechanism classification

Injury mechanism was categorized based on clinical history and preoperative diagnosis. ‘Traumatic’ mechanisms included acute injuries resulting from specific events: traffic accidents (motor vehicle collisions), falls (from standing height or greater), and sports-related sprains (sudden twisting or impact during athletic activity). ‘Degenerative’ mechanisms included insidious onset of symptoms without identifiable acute injury, typically in older patients with radiographic evidence of osteoarthritis or degenerative meniscal tears. This classification was determined by the treating surgeon based on patient history and recorded in the medical record.

### Data collection

Demographic, surgical, and laboratory data were collected. HBL was calculated using the Gross formula. Inflammatory markers (CRP and WBC) were measured preoperatively and within 48 h postoperatively.

### Blood sample collection and processing

Venous blood samples were collected from all patients within 24 h preoperatively and again within 48 h postoperatively. Samples were drawn into EDTA tubes for complete blood count analysis and into serum separator tubes for CRP measurement. All samples were processed within 2 h of collection by the hospital’s central laboratory. Complete blood counts were analyzed using an automated hematology analyzer (Sysmex XN-9000, Sysmex Corporation, Kobe, Japan). CRP was measured using immunoturbidimetric assay (Roche Cobas 8,000, Roche Diagnostics, Basel, Switzerland). Quality control was performed daily according to the manufacturer’s specifications. Laboratory personnel were blinded to the study objectives and patient clinical data.

### Calculation of hidden blood loss


$$\begin{array}{c} \text{Hidden Blood Loss}\;(\mathrm{mL})=\text{Total Blood Loss}\;(\mathrm{mL})\\ -\text{Visible Blood Loss}\;(\mathrm{mL})\\ +\text{Blood Transfusion Volume}\;(\mathrm{mL}) \end{array}$$

$$\begin{array}{c} \text{Total Blood Loss}\;(\mathrm{mL})=\text{Preoperative Blood Volume}\;(L)\\ \times [\begin{array}{l} \;\text{Preoperative Hematocrit}\;(\%)\\ -\text{Postoperative Hematocrit}\;(\%) \end{array}]\\ /\text{Average Hematocrit}\;(\%) \end{array}$$

Visible blood loss was measured using a standardized protocol. Intraoperative blood loss was quantified by measuring the volume in the suction canister after subtracting the estimated irrigation fluid used during the procedure. Any blood on surgical drapes, sponges, and gauze was estimated by weighing these materials immediately before and after use (1 g weight increase = 1 mL blood loss). In addition, visible blood loss is calculated by adding the amount of blood in the postoperative drainage tube.

### Average hematocrit calculation

The average hematocrit used in the Gross formula was calculated as the arithmetic mean of preoperative hematocrit values.

Estimated preoperative blood volume (EBV) for each patient was calculated using the Nadler formula (4). EBV(L) = k1 × height(m)^3^ + k2 × weight(kg) + k3. For males: k1 = 0.3669, k2 = 0.03219, k3 = 0.6041; for females: k1 = 0.3561, k2 = 0.03308, k3 = 0.1833.

### Statistical analysis

Based on previous research (9) which identified gender, preoperative hematocrit, postoperative hematocrit, and preoperative blood volume as independent factors associated with HBL, this study considered only age, gender, preoperative hematocrit, postoperative hematocrit, and preoperative blood volume as covariates.

Continuous variables are described as mean ± standard deviation (X ± SD) or median (interquartile range), M (IQR). Categorical variables are described as frequency (%). ∆CRP = Postoperative CRP—Preoperative CRP, ∆WBC = Postoperative WBC—Preoperative WBC. Linear regression was used to analyze the association between HBL and ∆CRP/∆WBC. SPSS 26.0 was used for data analysis. A *p*-value < 0.05 was considered statistically significant.

For comparing preoperative and postoperative CRP and WBC, paired t-tests were used if the differences were normally distributed; otherwise, paired Wilcoxon signed-rank tests were used. Factors influencing changes in CRP and WBC were further analyzed. Changes were calculated as: ∆CRP = Postoperative CRP—Preoperative CRP, ∆WBC = Postoperative WBC—Preoperative WBC. If the residuals of ∆CRP or ∆WBC were normally distributed, linear regression was used; if not, BOX-COX transformation was applied to achieve normality. SPSS 26.0 was used for data analysis. A *p*-value < 0.05 was considered statistically significant.

### Statistical power analysis

A post-hoc power analysis was conducted using G*Power software (version 3.1.9.7). With a sample size of 34, *α* = 0.05, and including 7 predictors in the multivariate model, the study had 80% power to detect a large effect size (f^2^ ≥ 0.35, equivalent to R^2^ ≥ 0.26) for the association between HBL and ∆CRP/∆WBC. The study was underpowered to detect small-to-moderate effects.

## Results

### Baseline characteristics for hidden blood loss

A total of 34 patients were included. The mean age of the entire cohort was 45.47 ± 17.20 years, including 12 males (35.29%; Table 1).

**Table 1:** Basic characteristics of the study population (hidden blood loss).

Characteristic, Mean ± SD	All patients (*N* = 34)
Age (years)	45.47 ± 17.20
Gender, Male, n (%)	12.00 ± 35.29
Preoperative Hematocrit (%), M (IQR)	43.30 (40.05, 45.85)
Postoperative Hematocrit (%)	38.35 ± 5.37
Preoperative Blood Volume (L)	6.16 ± 1.09
∆WBC (x10^9^/L)	2.87 ± 1.29
∆CRP (mg/L), M (IQR)	3.59 (1.67, 7.65)
Hidden Blood Loss (ml), M (IQR)	393.60 (273.42, 672.97)

Data are presented as mean ± SD unless otherwise specified. M (IQR), Median (Interquartile Range).

### Association between inflammatory markers and hidden blood loss

The associations between ∆CRP/∆WBC and hidden blood loss were not statistically significant (*p* > 0.05; Table 2).

**Table 2:** Analysis of factors influencing hidden blood loss.

Factors	Univariate analysis *β* (95% CI)	Multivariate analysis β (95% CI)
Age	0.470 (−7.021–7.960)	−1.033 (−5.113–3.048)
Gender	−173.041 (−431.296–85.214)	−94.909 (−315.566–125.748)
Preoperative hematocrit (%)	6.346 (−17.866–30.558)	**126.579 (100.413**–152.745)**
Postoperative hematocrit (%)	−19.170 (−42.170–3.830)	**−116.322 (−141.372**–−91.271)**
Preoperative blood volume (L)	98.994 (−13.471–211.460)	**131.857 (44.937**–218.776)**
∆CRP	−3.351 (−11.074–4.372)	−0.131 (−50.116–49.855)
∆WBC	−34.183 (−133.565–65.200)	0.762 (−3.132–4.656)

*p < 0.05; ** *p* < 0.01. Bold values indicate statistical significance in the multivariate analysis, showing that preoperative hematocrit, postoperative hematocrit, and preoperative blood volume are independently associated with hidden blood loss.

### Changes in inflammatory markers before and after surgery

The median postoperative CRP was 5.36 (IQR 2.80–12.20), compared to a preoperative median of 1.08 (IQR 0.60–2.40). The difference between postoperative and preoperative CRP was statistically significant (*p* = 0.000). The mean postoperative WBC was 9.25 ± 2.07, compared to a preoperative mean of 6.38 ± 2.06. The difference between postoperative and preoperative WBC was statistically significant (*p* = 0.000; Table 3).

**Table 3:** Comparison of preoperative and postoperative CRP and WBC.

Time Point	CRP		WBC	
	Median	Quartile	Mean	SD
preoperative	1.08	0.60–2.40	6.38	2.06
postoperative	5.36	2.80–12.20	9.25	2.07
Z/t	−5.086		13.021	
*p*	0.000**		0.000**	

*p < 0.05; **p < 0.01. Bold p-values indicate a statistically significant increase in both CRP and WBC from preoperative to postoperative measurements.

### Baseline characteristics for inflammatory markers

A total of 34 patients were included. The mean age of the entire cohort was 45.47 ± 17.20 years, including 12 males (35.29%; Table 4).

**Table 4:** Basic characteristics of the study population (inflammatory markers).

Characteristic	All patients (*N* = 34)
Age (years)	45.47 ± 17.20
Gender, Male, n (%)	12.00 ± 35.29
BMI(Kg/m^2^)	25.94 ± 4.01
Operation Time (min), M (IQR)	80.00 (58.75, 92.50)
Hospital Stay (days), M (IQR)	6.00 (5.00, 8.25)
Preoperative RBC (10^12^/L)	4.60 ± 0.48
Preoperative Hemoglobin (g/L), M (IQR)	142.50 (130.25, 150.00)
Preoperative Hematocrit (%), M (IQR)	43.30 (40.05, 45.85)
Postoperative RBC (10^12^/L)	4.27 ± 0.48
Postoperative Hemoglobin (g/L), M (IQR)	129.50 (116.25, 141.25)
Postoperative Hematocrit (%), M (IQR)	38.65 (34.65, 42.93)
Preoperative Blood Volume (L)	6.16 ± 1.09
Total Blood Loss (ml), M (IQR)	444.36 (292.66, 775.10)
Hidden Blood Loss (ml), M (IQR)	393.60 (273.42, 672.97)
Comorbidities, Yes, n (%)	7(20.59)
Injury Mechanism, n (%)	
Traffic Accident, Fall, Sprain	26(76.47)
Degeneration	8(23.53)

Data are presented as mean ± SD unless otherwise specified. M (IQR), Median (Interquartile Range). RBC, Red Blood Cell count. Hospital stay reflects regional practice patterns including inpatient stay until suture removal.

### Factors associated with inflammatory markers

Neither univariate nor multivariate analysis showed statistically significant associations between the examined factors and increases in CRP or WBC (*p* > 0.05; Table 5).

**Table 5:** Analysis of factors influencing increases in CRP and WBC.

Variable	∆CRP, β (95% CI)		∆WBC, β (95% CI)	
	Univariate	Multivariate	Univariate	Multivariate
Age	0.010 (−0.013–0.033)	0.009 (−0.030–0.049)	−0.009 (−0.036–0.018)	−0.010 (−0.056–0.037)
Gender	0.155 (−0.671–0.982)	−0.583 (−3.149–1.984)	0.157 (−0.797–1.110)	−0.641 (−3.681–2.399)
BMI(Kg/m^2^)	0.016 (−0.084–0.116)	0.104 (−0.085–0.293)	−0.064 (−0.177–0.049)	−0.076 (−0.299–0.148)
Operation Time (min)	0.003 (−0.008–0.013)	0.003 (−0.014–0.021)	0.002 (−0.010–0.014)	0.006 (−0.015–0.026)
Hospital Stay (days)	0.040 (−0.040–0.121)	0.123 (−0.010–0.257)	−0.003 (−0.097–0.092)	−0.043 (−0.201–0.116)
Preop. RBC (10^12^/L)	−0.078 (−0.907–0.751)	5.297 (−5.815–16.410)	0.229 (−0.724–1.182)	1.554 (−11.608–14.716)
Preop. Hemoglobin (g/L)	−0.002 (−0.023–0.019)	−0.407 (−0.946–0.132)	0.015 (−0.008–0.039)	0.127 (−0.511–0.766)
Preop. Hematocrit (%)	−0.008 (−0.084–0.067)	0.244 (−1.668–2.155)	0.044 (−0.042–0.130)	0.184 (−2.080–2.448)
Postop. RBC (10^12^/L)	0.157 (−0.684–0.998)	−5.633 (−17.516–6.251)	0.379 (−0.584–1.341)	−1.387 (−15.463–12.688)
Postop. Hemoglobin (g/L)	0.003 (−0.019–0.026)	0.364 (−0.110–0.837)	0.020 (−0.005–0.045)	0.036 (−0.525–0.597)
Postop. Hematocrit (%)	−0.006 (−0.081–0.069)	0.001 (−1.534–1.536)	0.054 (−0.030–0.138)	−0.681 (−2.500–1.137)
Preop. Blood Volume (L)	0.017 (−0.351–0.385)	−0.450 (−1.854–0.953)	−0.198 (−0.616–0.221)	0.344 (−1.319–2.006)
Total Blood Loss (ml)	0.000 (−0.001–0.001)	0.002 (−0.013–0.017)	−0.001 (−0.002–0.001)	−0.008 (−0.026–0.010)
Hidden Blood Loss (ml)	0.000 (−0.001–0.001)	0.001 (−0.006–0.008)	−0.000 (−0.002–0.001)	0.003 (−0.006–0.011)
Comorbidities (Yes)	0.339 (−0.632–1.311)	−0.860 (−2.342–0.622)	0.555 (−0.556–1.666)	0.766 (−0.989–2.521)
Injury Mechanism	−0.447 (−1.366–0.472)	−0.417 (−2.038–1.204)	0.390 (−0.677–1.457)	0.466 (−1.454–2.386)

## Discussion

This retrospective cohort study of 34 patients undergoing knee arthroscopy yielded two main findings. First, while both CRP and WBC increased significantly postoperatively (*p* < 0.001), neither ∆CRP nor ∆WBC was associated with HBL in univariate or multivariate analyses. Second, consistent with the mathematical derivation of HBL, preoperative hematocrit, postoperative hematocrit, and estimated blood volume were significant factors in the HBL calculation. These findings suggest that postoperative bacterial inflammation and hidden blood loss represent distinct pathophysiological processes following knee arthroscopy.

### Clinical interpretation

The absence of an association between HBL and inflammatory markers has important clinical implications. In postoperative orthopedic patients, elevated CRP and WBC often raise concern for infection, potentially leading to unnecessary diagnostic testing, prolonged hospital stays, or even empirical antibiotic therapy (13). Our results suggest that in the setting of knee arthroscopy, the degree of inflammatory marker elevation is independent of the amount of hidden blood loss. Conversely, significant HBL should not be interpreted as a risk factor for infection, nor should it alter infection surveillance strategies. The median HBL in our study was 393.60 mL (IQR 273.42–672.97), which is substantial for a procedure often considered ‘minimally invasive.’ This finding aligns with growing evidence that HBL is clinically significant across various arthroscopic procedures, although direct comparisons are limited by differing surgical techniques and patient populations. Matejcic et al. (4) recently reported a mean HBL of 754.72 mL in shoulder arthroscopy, while Chen and Peng (3) found average HBL of 287.2 mL in percutaneous vertebroplasty. In the knee arthroscopy literature, our previous work (9) demonstrated similar HBL ranges to the current study. These cumulative data, despite coming from relatively small studies, consistently suggest that surgeons should maintain vigilance for postoperative anemia even after seemingly bloodless procedures. However, given the small sample size of the current study, these findings should be considered hypothesis-generating rather than definitive, and larger multicenter studies are needed to establish more precise estimates of HBL and its clinical significance across different arthroscopic procedures.

### Pathophysiological interpretation

The lack of association between HBL and bacterial inflammatory markers (CRP, WBC) can be explained by considering the distinct mechanisms underlying these phenomena. HBL after knee arthroscopy primarily results from three mechanisms: (1) extravasation of blood into the joint cavity and periarticular tissues, (2) hemolysis related to tourniquet use and irrigation fluid pressure, and (3) postoperative bleeding into tissue planes (9). The use of motorized shavers for synovectomy, meniscectomy, or ligament debridement can damage nearby vascular structures (14), while tourniquet-induced ischemia followed by reperfusion may trigger hemolysis through oxidative stress pathways (15). In contrast, postoperative elevations in CRP and WBC reflect the systemic inflammatory response to surgical trauma. This response is mediated by cytokines such as IL-6, which stimulates hepatic CRP production, and by the release of neutrophils from bone marrow stores (16). Importantly, the inflammatory markers measured in routine clinical practice (CRP, WBC) are primarily indicators of bacterial inflammation and may not capture the sterile inflammatory response triggered by tissue damage and oxidative stress (17). Markers such as IL-1β, TNF-*α*, and COX-2 are more specific to sterile inflammation (18) and were not measured in our study. Therefore, while HBL and sterile inflammation may be related through shared oxidative stress pathways, this relationship would not be detected using routine bacterial inflammatory markers. After prolonged tissue ischemia, compensatory increase in anaerobic metabolism activates a large number of oxygen free radicals, placing the body in a state of oxidative stress (15). Oxidative stress can damage red blood cell membranes, inducing intravascular hemolysis (19), and hemolysis can further activate oxidative stress through release of free hemoglobin and iron (20), potentially creating a self-perpetuating cycle that contributes to HBL.

### Comparison with previous studies

Our finding that preoperative hematocrit and blood volume are mathematically related to HBL is consistent with previous work, including our own (9). Similarly, studies in hip fracture surgery (21) have demonstrated that a greater hemoglobin drop during the perioperative period is associated with greater calculated HBL. This mathematical relationship is inherent to the Gross formula and serves primarily as a validation of our calculation methodology rather than a novel discovery. The novel contribution of our study is the demonstration that HBL and bacterial inflammatory markers are independent phenomena, which has not been previously reported in the knee arthroscopy literature.

### Limitations

Several limitations of this study must be acknowledged. First, the sample size (N = 34) is relatively small, which limits the statistical power to detect modest associations and increases the risk of type II error. While our post-hoc power analysis indicated 80% power to detect a large effect size (r ≥ 0.45) for the association between HBL and ∆CRP, smaller but potentially clinically relevant effects may have been missed. Second, the single-center design and specific patient population may limit generalizability to other settings or surgical techniques. Future multicenter studies with larger sample sizes are needed to confirm our findings and to enable subgroup analyses based on specific procedures (e.g., ACL reconstruction vs. simple meniscectomy), which may have different HBL and inflammatory profiles. Such studies should also include longer follow-up periods to assess clinical outcomes such as infection rates and functional recovery. This study did not include markers of sterile inflammation for comparison, which warrants investigation in future research.

## Conclusion

This study demonstrates that while significant increases in bacterial inflammatory markers (CRP and WBC) occur after knee arthroscopy, these changes are independent of hidden blood loss. The median HBL of approximately 400 mL, though substantial for a minimally invasive procedure, does not correlate with the degree of inflammatory response. These findings have two important clinical implications: First, postoperative elevations in CRP and WBC should be interpreted as expected physiological responses to surgical trauma rather than indicators of excessive bleeding or infection risk related to blood loss. Second, strategies to minimize HBL—primarily through optimization of preoperative hematocrit—should focus on preventing postoperative anemia rather than modifying inflammatory responses. Future studies incorporating markers of sterile inflammation (e.g., IL-1β, TNF-*α*) may further elucidate whether HBL relates to non-bacterial inflammatory pathways.

## Data Availability

The datasets presented in this study can be found in online repositories. The names of the repository/repositories and accession number(s) can be found in the article/Supplementary material.
