# Supplementary material for: Postoperative inflammatory markers are not associated with hidden blood loss after knee arthroscopy
Source: Front Med (Lausanne). 2026 Mar 17;13:1783296. doi: 10.3389/fmed.2026.1783296 (PMC13041567; doi:10.3389/fmed.2026.1783296)
Supplement: Supplementary file 1 [file Data_Sheet_1.zip › 表1描述性分析结果.pdf]

基础指标 排序

名称	样本量	最小值	最大值	平均值	标准差	中位数
年龄	34	17.000	74.000	45.471	17.200	45.500
性别	34	1.000	2.000	1.353	0.485	1.000
BMI(KG/M2)	34	18.591	38.580	25.941	4.012	25.460
手术时间（分钟）	34	20.000	170.000	80.912	38.566	80.000
住院时间（天）	34	3.000	32.000	7.412	4.906	6.000
术前红细胞*10（12）/L	34	3.350	5.550	4.604	0.484	4.635
术前血红蛋白g/L	34	83.000	171.000	138.206	19.042	142.500
术前红细胞压体积%	34	28.600	51.300	42.441	5.299	43.300
术后红细胞*10（12）/L	34	3.400	5.090	4.268	0.477	4.370
术后血红蛋白g/L	34	78.000	156.000	127.471	17.865	129.500
术后红细胞压体积%	34	25.900	47.900	38.350	5.366	38.650
术前血容量（L）	34	4.128	7.753	6.161	1.092	6.264
实际失血总量（ml）	34	116.464	1441.226	584.995	365.912	444.358
隐性失血	34	48.088	1436.226	524.112	357.881	393.595

分析建议

描述分析用于研究定量数据的整体情况，整体平均得分情况如何等；
第一：整体描述分析项平均得分值情况；
第二：着重对平均值较高，或者明显较低分析项进行说明；
第三：如果标准差值较大，可考虑使用中位数表示整体打分情况；
第四：对分析进行总结。
另外SPSSAU还提供比如方差，分位数，偏度和峰度等指标。

智能分析

描述性分析通过平均值或中位数描述数据的整体情况。从上表可以看出：BMI(KG/M2), 住院时间（天）共2项的最大值超过平均值3个标准差【说明数据波动较大，相对平均值，使用中位数描述整体水平更适合】，SPSSAU建议使用中位数进行描述分析，而不是使用平均值。总结可知，BMI(KG/M2), 住院时间（天）共2项数据的最值(最小/最大值)超过平均值3个标准差【说明数据波动较大，相对平均值，使用中位数描述整体水平更适合】，SPSSAU建议使用中位数进行描述分析，而不是使用平均值。以及如果需要更多详细细节指标请参考下表。

折线 柱形 条形 雷达

平均值对比图

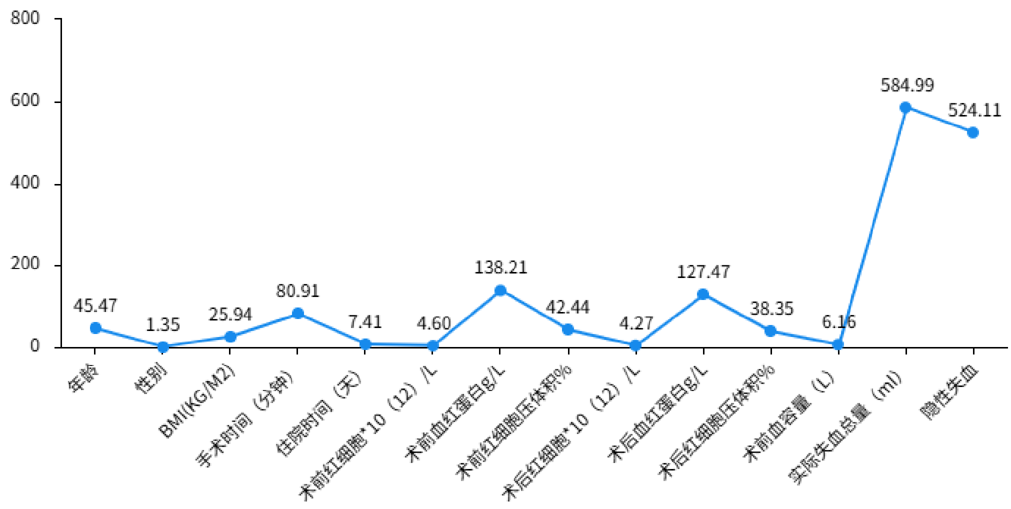

复制 下载 排序 尺寸： 样式

深入指标

名称	平均值±标准差	方差	求和	25分位数	中位数	75分位数	标准误	均值95% CI(*LL*)	均值95% CI(*UL*)	IQR	峰度	偏度	变异系数(CV)
年龄	45.471±17.200	295.832	1546.000	31.000	45.500	61.000	2.950	39.689	51.252	30.000	-1.031	-0.104	37.826%
性别	1.353±0.485	0.235	46.000	1.000	1.000	2.000	0.083	1.190	1.516	1.000	-1.688	0.644	35.853%
BMI(KG/M2)	25.941±4.012	16.096	881.984	23.434	25.460	28.345	0.688	24.592	27.289	4.911	2.178	0.881	15.466%
手术时间（分钟）	80.912±38.566	1487.356	2751.000	58.750	80.000	92.500	6.614	67.948	93.875	33.750	0.058	0.709	47.665%

住院时间（天）	7.412±4.906	24.068	252.000	5.000	6.000	8.250	0.841	5.763	9.061	3.250	19.771	4.004	66.191%
术前红细胞*10（12）/L	4.604±0.484	0.235	156.540	4.305	4.635	5.025	0.083	4.441	4.767	0.720	0.148	-0.426	10.523%
术前血红蛋白g/L	138.206±19.042	362.593	4699.000	130.250	142.500	150.000	3.266	131.805	144.606	19.750	1.676	-1.162	13.778%
术前红细胞压体积%	42.441±5.299	28.077	1443.000	40.050	43.300	45.850	0.909	40.660	44.222	5.800	0.946	-0.934	12.485%
术后红细胞*10（12）/L	4.268±0.477	0.227	145.110	3.790	4.370	4.683	0.082	4.108	4.428	0.893	-1.174	-0.106	11.168%
术后血红蛋白g/L	127.471±17.865	319.166	4334.000	116.250	129.500	141.250	3.064	121.466	133.476	25.000	0.915	-0.911	14.015%
术后红细胞压体积%	38.350±5.366	28.798	1303.900	34.650	38.650	42.925	0.920	36.546	40.154	8.275	-0.146	-0.526	13.993%
术前血容量（L）	6.161±1.092	1.193	209.462	5.513	6.264	7.053	0.187	5.793	6.528	1.540	-0.852	-0.353	17.730%
实际失血总量（ml）	584.995±365.912	133891.897	19889.817	292.656	444.358	775.102	62.753	462.000	707.989	482.447	0.007	0.971	62.550%
隐性失血	524.112±357.881	128078.518	17819.817	273.419	393.595	672.970	61.376	403.817	644.407	399.551	0.469	1.080	68.283%

百分位数

名称	P2.5	P5	P10	P25	P27	P33	P50	P67	P73	P75	P90	P95	P97.5
年龄	17.000	17.000	18.000	31.000	32.450	36.100	45.500	55.800	59.650	61.000	69.000	73.250	74.000
性别	1.000	1.000	1.000	1.000	1.000	1.000	1.000	2.000	2.000	2.000	2.000	2.000	2.000
BMI(KG/M2)	18.591	19.003	20.595	23.434	23.634	24.524	25.460	27.504	28.046	28.345	30.518	35.365	38.580
手术时间（分钟）	20.000	23.750	33.000	58.750	60.000	60.000	80.000	90.000	90.000	92.500	150.000	162.500	170.000
住院时间（天）	3.000	3.750	4.000	5.000	5.000	5.000	6.000	8.000	8.000	8.250	10.500	17.000	32.000
术前红细胞*10（12）/L	3.350	3.710	3.900	4.305	4.353	4.471	4.635	4.810	4.966	5.025	5.205	5.408	5.550
术前血红蛋白g/L	83.000	91.250	108.500	130.250	132.000	134.100	142.500	148.450	149.550	150.000	159.000	165.000	171.000
术前红细胞压体积%	28.600	30.175	33.850	40.050	40.780	41.685	43.300	44.535	45.665	45.850	48.550	50.475	51.300
术后红细胞*10（12）/L	3.400	3.558	3.640	3.790	3.833	3.948	4.370	4.550	4.662	4.683	4.890	5.060	5.090
术后血红蛋白g/L	78.000	83.250	105.000	116.250	117.450	119.000	129.500	138.000	140.550	141.250	147.500	151.500	156.000
术后红细胞压体积%	25.900	26.875	30.300	34.650	34.745	35.595	38.650	41.800	42.630	42.925	44.500	46.025	47.900
术前血容量（L）	4.128	4.213	4.329	5.513	5.693	5.780	6.264	6.727	6.917	7.053	7.657	7.745	7.753
实际失血总量（ml）	116.464	160.257	212.498	292.656	302.274	350.257	444.358	654.407	723.721	775.102	1292.509	1348.792	1441.226
隐性失血	48.088	84.370	156.780	273.419	281.143	314.671	393.595	592.586	627.475	672.970	1215.806	1305.096	1436.226

缺失分析

名称	样本量	缺失样本量	最小值	最大值	平均值	标准差	中位数	是否数字恒定
年龄	34	0	17.000	74.000	45.471	17.200	45.500	否
性别	34	0	1.000	2.000	1.353	0.485	1.000	否
BMI(KG/M2)	34	0	18.591	38.580	25.941	4.012	25.460	否
手术时间（分钟）	34	0	20.000	170.000	80.912	38.566	80.000	否
住院时间（天）	34	0	3.000	32.000	7.412	4.906	6.000	否
术前红细胞*10（12）/L	34	0	3.350	5.550	4.604	0.484	4.635	否
术前血红蛋白g/L	34	0	83.000	171.000	138.206	19.042	142.500	否
术前红细胞压体积%	34	0	28.600	51.300	42.441	5.299	43.300	否
术后红细胞*10（12）/L	34	0	3.400	5.090	4.268	0.477	4.370	否
术后血红蛋白g/L	34	0	78.000	156.000	127.471	17.865	129.500	否
术后红细胞压体积%	34	0	25.900	47.900	38.350	5.366	38.650	否
术前血容量（L）	34	0	4.128	7.753	6.161	1.092	6.264	否
实际失血总量（ml）	34	0	116.464	1441.226	584.995	365.912	444.358	否
隐性失血	34	0	48.088	1436.226	524.112	357.881	393.595	否

备注：全局过滤后样本量 = 34

分析建议

缺失分析表格展示各分析项的缺失情况；

第一：'样本量'指某分析项独立分析时的样本量；

第二：'缺失样本量'指该某分析项独立分析时的缺失样本量；

第三：'全局过滤后样本量'指全部分析项均有完整数据时的样本量；

第四：'全局过滤后样本量'一定会小于等于各分析项的样本量；

第五：如果某项时最小值与最大值完全相同，则说明其恒定为一个固定数字。
