# Supplementary material for: Postoperative inflammatory markers are not associated with hidden blood loss after knee arthroscopy
Source: Front Med (Lausanne). 2026 Mar 17;13:1783296. doi: 10.3389/fmed.2026.1783296 (PMC13041567; doi:10.3389/fmed.2026.1783296)
Supplement: Supplementary file 1 [file Data_Sheet_1.zip › 表2 配对t检验.pdf]

配对*t* 检验分析结果

名称	配对(平均值±标准差)		差值(配对1-配对2)	*t*	*p*
	配对1	配对2			
术后白细胞10（9）/L *配对* 术前白细胞10（9）/L	9.25±2.07	6.38±2.06	2.87	13.021	0.000**

* $p<0.05$ ** $p<0.01$

分析建议

配对*t*检验研究配对数据的差异关系；
第一：分析每组配对项之间是否呈现出显著性差异(*p*值小于0.05或0.01)；
第二：如果呈现出显著性；具体对比平均值(或差值)大小，描述具体差异所在；
第三：对分析进行总结。

智能分析

从上表可知，利用配对t检验去研究实验数据的差异性，从上表可以看出：总共1组配对数据，均会呈现出差异性($p<0.05$) 。具体分析可知：
术后白细胞10（9）/L和术前白细胞10（9）/L之间呈现出0.01水平的显著性($t=13.021$，$p=0.000$)，以及具体对比差异可知，术后白细胞10（9）/L的平均值(9.25)，会明显高于术前白细胞10（9）/L的平均值(6.38)。
总共1组配对数据全部均会呈现出差异性。

深入分析-效应量指标

名称	平均值差值	差值95% CI	*df*	差值标准差	Cohen's *d* 值
术后白细胞10（9）/L *配对* 术前白细胞10（9）/L	2.87	2.425 ~ 3.323	33	1.287	2.233

分析建议

如果显示呈现出显著性差异($p<0.05$)，可通过平均值对比具体差异，同时还可使用效应量(Effect size)研究差异幅度情况；
第一：使用Cohen's *d* 值表示效应量大小(差异幅度大小)，该值越大说明差异越大；
第二：配对样本*t*检验使用Cohen's *d* 值表示效应量大小时， 效应量小、中、大的区分临界点分别是: 0.20、0.50和0.80；
第三：Cohen's *d* 值计算公式为差值的绝对值/标准差。

配对*t* 检验分析结果-详细格式

配对编号	项	平均值	标准差	平均值差值	*t*	*p*
配对1	术后白细胞10（9）/L	9.25	2.07	2.87	13.021	0.000**
	术前白细胞10（9）/L	6.38	2.06			

* $p<0.05$ ** $p<0.01$

参考文献

【1】The SPSSAU project (2025). SPSSAU. (Version 25.0) [Online Application Software]. Retrieved from https://www.spssau.com.
【2】周俊,马世澎. SPSSAU科研数据分析方法与应用.第1版[M]. 电子工业出版社,2024.
【3】张厚粲, 徐建平. 现代心理与教育统计学.第3版[M]. 北京师范大学出版社, 2009.
【4】颜虹,徐勇勇. 医学统计学.第3版[M].人民卫生出版社,2017.
