# Supplementary material for: Postoperative inflammatory markers are not associated with hidden blood loss after knee arthroscopy
Source: Front Med (Lausanne). 2026 Mar 17;13:1783296. doi: 10.3389/fmed.2026.1783296 (PMC13041567; doi:10.3389/fmed.2026.1783296)
Supplement: Supplementary file 1 [file Data_Sheet_1.zip › 表3 CRP增高影响因素分析.pdf]

线性回归分析结果 (n=34)

	非标准化系数		标准化系数	t	p	共线性诊断	
	B	标准误	$Beta$			VIF	容忍度
常数	-1.513	6.555	-	-0.231	0.820	-	-
年龄	0.009	0.019	0.140	0.484	0.634	2.372	0.422
性别	-0.583	1.216	-0.253	-0.479	0.638	7.960	0.126
BMI(KG/M2)	0.104	0.090	0.375	1.163	0.261	2.950	0.339
手术时间（分钟）	0.003	0.008	0.115	0.404	0.691	2.309	0.433
住院时间（天）	0.123	0.063	0.541	1.947	0.068	2.202	0.454
术前红细胞*10（12）/L	5.297	5.267	2.300	1.006	0.329	148.865	0.007
术前血红蛋白g/L	-0.407	0.256	-6.945	-1.593	0.130	541.292	0.002
术前红细胞压体积%	0.244	0.906	1.158	0.269	0.791	526.955	0.002
术后红细胞*10（12）/L	-5.633	5.633	-2.406	-1.000	0.331	164.794	0.006
术后血红蛋白g/L	0.364	0.224	5.825	1.621	0.123	367.443	0.003
术后红细胞压体积%	0.001	0.728	0.005	0.001	0.999	348.595	0.003
术前血容量（L）	-0.450	0.665	-0.441	-0.677	0.507	12.071	0.083
实际失血总量（ml）	0.002	0.007	0.604	0.260	0.798	153.594	0.007
隐性失血（ml）	0.001	0.003	0.392	0.356	0.726	34.439	0.029
是否内科病	-0.860	0.702	-0.316	-1.224	0.237	1.900	0.526
损伤机制	-0.417	0.768	-0.161	-0.542	0.595	2.502	0.400
R^2	0.403						
调整R^2	-0.159						
F	F (16,17)=0.716,p=0.745						
D-W值	1.540						

备注：因变量 = BoxCox_-0.02384337832845352_CRP增高值

* p<0.05 ** p<0.01

分析建议

回归分析用于研究X(定量或定类)对Y(定量)的影响关系，是否有影响关系，影响方向及影响程度情况如何；

第一：首先分析模型拟合情况，即通过R方值分析模型拟合情况，以及可对VIF值进行分析（或者容忍度值，容忍度=1/VIF值，VIF值一般>5说明有共线性问题，容忍度一般<0.2说明有共线性问题），判断模型是否存在共线性问题【共线性问题可使用岭回归或者逐步回归进行解决】；

第二：写出模型公式(可选)；

第三：分析X的显著性；如果呈现出显著性(p值小于0.05或0.01)；则说明X对Y有影响关系，接着具体分析影响关系方向；

第四：结合回归系数B值，对比分析X对Y的影响程度(可选)；

第五：对分析进行总结。

回归分析之前，可使用箱盒图查看是否有异常数据，或使用散点图直观展示X和Y之间的关联关系；回归分析之后，可使用正态图观察和展示保存的残差值正态性情况；或使用散点图观察和展示回归模型异方差情况【残差与X间的散点完全没有关系则无异方差】。

智能分析

从上表可知，将年龄,性别,BMI(KG/M2),手术时间（分钟）,住院时间（天）,术前红细胞*10（12）/L,术前血红蛋白g/L,术前红细胞压体积%,术后红细胞*10（12）/L,术后血红蛋白g/L,术后红细胞压体积%,术前血容量（L）,实际失血总量（ml）,隐性失血（ml）,是否内科病,损伤机制作为自变量，而将BoxCox_-0.02384337832845352_CRP增高值作为因变量进行线性回归分析，从上表可以看出，模型公式为：BoxCox_-0.02384337832845352_CRP增高值=-1.513 + 0.009*年龄-0.583*性别 + 0.104*BMI(KG/M2) + 0.003*手术时间（分钟） + 0.123*住院时间（天） + 5.297*术前红细胞*10（12）/L-0.407*术前血红蛋白g/L + 0.244*术前红细胞压体积%-5.633*术后红细胞*10（12）/L + 0.364*术后血红蛋白g/L + 0.001*术后红细胞压体积%-0.450*术前血容量（L） + 0.002*实际失血总量（ml） + 0.001*隐性失血（ml）-0.860*是否内科病-0.417*损伤机制，模型R方值为0.403，意味着年龄,性别,BMI(KG/M2),手术时间（分钟）,住院时间（天）,术前红细胞*10（12）/L,术前血红蛋白g/L,术前红细胞压体积%,术后红细胞*10（12）/L,术后血红蛋白g/L,术后红细胞压体积%,术前血容量（L）,实际失血总量（ml）,隐性失血（ml）,是否内科病,损伤机制可以解释BoxCox_-0.02384337832845352_CRP增高值的40.3%变化原因。对模型进行F检验时发现模型并没有通过F检验(F=0.716，p=0.745>0.05)，也即说明年龄,性别,BMI(KG/M2),手术时间（分钟）,住院时间（天）,术前红细胞*10（12）/L,术前血红蛋白g/L,术前红细胞压体积%,术后红细胞*10（12）/L,术后血红蛋白g/L,术后红细胞压体积%,术前血容量（L）,实际失血总量（ml）,隐性失血（ml）,是否内科病,损伤机制并不会对BoxCox_-0.02384337832845352_CRP增高值产生影响关系，因而不能具体分析自变量对于因变量的影响关系，分析结束。

模型预测

BoxCox_-0.02384337832845352_CRP增高值	=	-1.5131927477
年龄	=	输入数字
性别	=	输入数字
BMI(KG/M2)	=	输入数字
手术时间（分钟）	=	输入数字
住院时间（天）	=	输入数字
术前红细胞*10（12）/L	=	输入数字
术前血红蛋白g/L	=	输入数字
术前红细胞压体积%	=	输入数字
术后红细胞*10（12）/L	=	输入数字
术后血红蛋白g/L	=	输入数字
术后红细胞压体积%	=	输入数字
术前血容量（L）	=	输入数字
实际失血总量（ml）	=	输入数字
隐性失血（ml）	=	输入数字
是否内科病	=	输入数字
损伤机制	=	输入数字

线性回归分析结果-简化格式

	回归系数	95% CI	共线性诊断	
			VIF	容忍度
常数	-1.513<br>(-0.231)	-15.343 ~ 12.316	-	-
年龄	0.009<br>(0.484)	-0.030 ~ 0.049	2.372	0.422
性别	-0.583<br>(-0.479)	-3.149 ~ 1.984	7.960	0.126
BMI(KG/M2)	0.104<br>(1.163)	-0.085 ~ 0.293	2.950	0.339
	0.003			

手术时间（分钟）	[illegible] (0.404)	-0.014 ~ 0.021	2.309	0.433
住院时间（天）	0.123 (1.947)	-0.010 ~ 0.257	2.202	0.454
术前红细胞*10（12）/L	5.297 (1.006)	-5.815 ~ 16.410	148.865	0.007
术前血红蛋白g/L	-0.407 (-1.593)	-0.946 ~ 0.132	541.292	0.002
术前红细胞压体积%	0.244 (0.269)	-1.668 ~ 2.155	526.955	0.002
术后红细胞*10（12）/L	-5.633 (-1.000)	-17.516 ~ 6.251	164.794	0.006
术后血红蛋白g/L	0.364 (1.621)	-0.110 ~ 0.837	367.443	0.003
术后红细胞压体积%	0.001 (0.001)	-1.534 ~ 1.536	348.595	0.003
术前血容量（L）	-0.450 (-0.677)	-1.854 ~ 0.953	12.071	0.083
实际失血总量（ml）	0.002 (0.260)	-0.013 ~ 0.017	153.594	0.007
隐性失血（ml）	0.001 (0.356)	-0.006 ~ 0.008	34.439	0.029
是否内科病	-0.860 (-1.224)	-2.342 ~ 0.622	1.900	0.526
损伤机制	-0.417 (-0.542)	-2.038 ~ 1.204	2.502	0.400
样本量		34		
R^2		0.403		
调整R^2		-0.159		
F值		F(16,17)=0.716,p=0.745		

备注：因变量 = BoxCox_-0.02384337832845352_CRP增高值

D-W值 = 1.540

* p<0.05 ** p<0.01 括号里面为t值

模型结果图

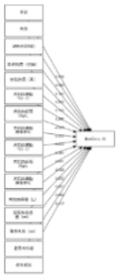

下载

展示数值

模型汇总(中间过程)

R	R^2	调整R^2	模型误差RMSE	DW值	AIC值	BIC值
0.635	0.403	-0.159	0.850	1.540	119.399	145.347

分析建议

回归分析用于研究X(定量或定类)对Y(定量)的影响关系，是否有影响关系，影响方向及影响程度情况如何；

第一：模型R方值分析模型拟合情况，比如0.5则表示模型有50%的拟合程度；

第二：调整R方值用于惩罚任意放置过多的X，通常情况下使用较少。

智能分析

从上表可知，将年龄,性别,BMI(KG/M2),手术时间（分钟）,住院时间（天）,术前红细胞*10（12）/L,术前血红蛋白g/L,术前红细胞压体积%,术后红细胞*10（12）/L,术后血红蛋白g/L,术后红细胞压体积%,术前血容量（L）,实际失血总量（ml）,隐性失血（ml）,是否内科病,损伤机制作为自变量，而将BoxCox_-0.02384337832845352_CRP增高值作为因变量进行线性回归分析，从上表可以看出，模型R方值为0.403，意味着年龄,性别,BMI(KG/M2),手术时间（分钟）,住院时间（天）,术前红细胞*10（12）/L,术前血红蛋白g/L,术前红细胞压体积%,术后红细胞*10（12）/L,术后血红蛋白g/L,术后红细胞压体积%,术前血容量（L）,实际失血总量（ml）,隐性失血（ml）,是否内科病,损伤机制可以解释BoxCox_-0.02384337832845352_CRP增高值的40.3%变化原因。

ANOVA表格(中间过程)

	平方和	df	均方	F	p 值
回归	16.545	16	1.034	0.716	0.745
残差	24.538	17	1.443		
总计	41.083	33			

分析建议

F检验用于检验回归模型是否有意义；

第一：如果模型通过F检验(p<0.05)，说明模型有意义，至少有一个X会对Y产生影响；

第二：如果模型没有通过F检验(p>0.05)，说明模型构建无意义，X均不会对Y产生影响。

智能分析

对模型进行F检验时发现模型并没有通过F检验(F=0.716，p=0.745>0.05)，也即说明年龄,性别,BMI(KG/M2),手术时间（分钟）,住院时间（天）,术前红细胞*10（12）/L,术前血红蛋白g/L,术前红细胞压体积%,术后红细胞*10（12）/L,术后血红蛋白g/L,术后红细胞压体积%,术前血容量（L）,实际失血总量（ml）,隐性失血（ml）,是否内科病,损伤机制并不会对BoxCox_-0.02384337832845352_CRP增高值产生影响关系，因而不能具体分析自变量对于因变量的影响关系。

回归系数(中间过程) (n=34)

	非标准化系数	标准化系数	[illegible]	[illegible]	[illegible]	[illegible]

	B	标准误	Beta	t	p	95% CI	VIF
常数	-1.513	6.555	-	-0.231	0.820	-15.343 ~ 12.316	-
年龄	0.009	0.019	0.140	0.484	0.634	-0.030 ~ 0.049	2.372
性别	-0.583	1.216	-0.253	-0.479	0.638	-3.149 ~ 1.984	7.960
BMI(KG/M2)	0.104	0.090	0.375	1.163	0.261	-0.085 ~ 0.293	2.950
手术时间（分钟）	0.003	0.008	0.115	0.404	0.691	-0.014 ~ 0.021	2.309
住院时间（天）	0.123	0.063	0.541	1.947	0.068	-0.010 ~ 0.257	2.202
术前红细胞*10（12）/L	5.297	5.267	2.300	1.006	0.329	-5.815 ~ 16.410	148.865
术前血红蛋白g/L	-0.407	0.256	-6.945	-1.593	0.130	-0.946 ~ 0.132	541.292
术前红细胞压体积%	0.244	0.906	1.158	0.269	0.791	-1.668 ~ 2.155	526.955
术后红细胞*10（12）/L	-5.633	5.633	-2.406	-1.000	0.331	-17.516 ~ 6.251	164.794
术后血红蛋白g/L	0.364	0.224	5.825	1.621	0.123	-0.110 ~ 0.837	367.443
术后红细胞压体积%	0.001	0.728	0.005	0.001	0.999	-1.534 ~ 1.536	348.595
术前血容量（L）	-0.450	0.665	-0.441	-0.677	0.507	-1.854 ~ 0.953	12.071
实际失血总量（ml）	0.002	0.007	0.604	0.260	0.798	-0.013 ~ 0.017	153.594
隐性失血（ml）	0.001	0.003	0.392	0.356	0.726	-0.006 ~ 0.008	34.439
是否内科病	-0.860	0.702	-0.316	-1.224	0.237	-2.342 ~ 0.622	1.900
损伤机制	-0.417	0.768	-0.161	-0.542	0.595	-2.038 ~ 1.204	2.502

备注：因变量 = BoxCox_-0.02384337832845352_CRP增高值

* $p<0.05$ ** $p<0.01$

coefPlot

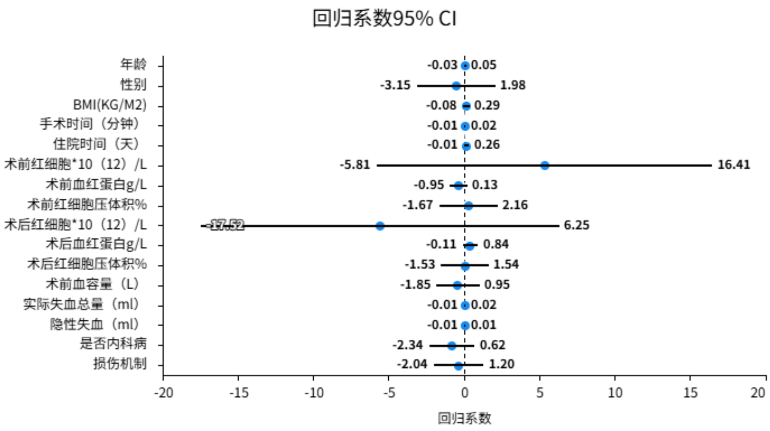

复制 下载 尺寸: 样式

样本缺失情况汇总

项	样本数	占比
有效样本	34	100.0%
排除无效样本	0	0.0%
总计	34	100%

分析建议

上表格展示真实进入算法模型时有效样本和排除在外的无效样本情况等。

第一：上表格中'有效样本'指所有分析项均有数据的样本总数，'排除无效样本'指任意一个分析项出现缺失的样本总数；

第二：如果某样本在任意一个分析项上出现缺失数据（即排除无效样本），该类样本无法进入模型分析，模型只能针对有效样本进行分析；

第三：可通过'通用方法'里面的描述分析检查各分析项样本情况，也可在右上角'查看数据'查看具体数据。

参考文献

【1】The SPSSAU project (2025). SPSSAU. (Version 25.0) [Online Application Software]. Retrieved from https://www.spssau.com.

【2】周俊,马世澎. SPSSAU科研数据分析方法与应用.第1版[M]. 电子工业出版社,2024.

【3】Sun Dao-de. Selection of the Linear Regression Model According to the Parameter Estimation[J]. Wuhan University Journal of Natural Sciences, 2000, 5(4):400-405.

【4】Barassi M R. Microeconometrics; Methods and Applications by A. Colin Cameron; Pravin K. Trivedi[J]. 2005.

【5】威廉H·格林.经济计量分析[M].北京:中国社会科学出版社,1998.

【6】张厚粲, 徐建平. 现代心理与教育统计学.第3版[M]. 北京师范大学出版社, 2009.
