# Supplementary material for: Postoperative inflammatory markers are not associated with hidden blood loss after knee arthroscopy
Source: Front Med (Lausanne). 2026 Mar 17;13:1783296. doi: 10.3389/fmed.2026.1783296 (PMC13041567; doi:10.3389/fmed.2026.1783296)
Supplement: Supplementary file 1 [file Data_Sheet_1.zip › 表5 隐性失血影响因素分析结果.pdf]

线性回归分析结果 (n=34)

	非标准化系数		标准化系数	t	p	共线性诊断	
	B	标准误	Beta			VIF	容忍度
常数	-1029.832	513.120	-	-2.007	0.055	-	-
年龄	-1.033	1.985	-0.050	-0.520	0.607	1.335	0.749
性别	-94.909	107.348	-0.129	-0.884	0.385	3.106	0.322
术前红细胞压体积%	126.579	12.729	1.874	9.944	0.000**	5.211	0.192
术后红细胞压体积%	-116.322	12.187	-1.744	-9.545	0.000**	4.899	0.204
术前血容量（L）	131.857	42.286	0.402	3.118	0.004**	2.444	0.409
白细胞增高值	-0.131	24.317	-0.000	-0.005	0.996	1.122	0.892
CRP增高值	0.762	1.894	0.035	0.402	0.691	1.117	0.895
R^2	0.823						
调整R^2	0.775						
F	$F(7,26)=17.243, p=0.000$						
D-W值	2.439						

备注：因变量 = 隐性失血（ml）

* $p<0.05$ ** $p<0.01$

分析建议

回归分析用于研究X(定量或定类)对Y(定量)的影响关系，是否有影响关系，影响方向及影响程度情况如何；

第一：首先分析模型拟合情况，即通过R方值分析模型拟合情况，以及可对VIF值进行分析（或者容忍度值，容忍度=1/VIF值，VIF值一般>5说明有共线性问题，容忍度一般<0.2说明有共线性问题），判断模型是否存在共线性问题【共线性问题可使用岭回归或者逐步回归进行解决】；

第二：写出模型公式(可选)；

第三：分析X的显著性；如果呈现出显著性(p值小于0.05或0.01)；则说明X对Y有影响关系，接着具体分析影响关系方向；

第四：结合回归系数B值，对比分析X对Y的影响程度(可选)；

第五：对分析进行总结。

回归分析之前，可使用箱盒图查看是否有异常数据，或使用散点图直观展示X和Y之间的关联关系；回归分析之后，可使用正态图观察和展示保存的残差值正态性情况；或使用散点图观察和展示回归模型异方差情况【残差与X间的散点完全没有关系则无异方差】。

智能分析

从上表可知，将年龄,性别,术前红细胞压体积%,术后红细胞压体积%,术前血容量（L）,白细胞增高值,CRP增高值作为自变量，而将隐性失血（ml）作为因变量进行线性回归分析，从上表可以看出，模型公式为：隐性失血（ml）=-1029.832-1.033*年龄-94.909*性别 + 126.579*术前红细胞压体积%-116.322*术后红细胞压体积% + 131.857*术前血容量（L）-0.131*白细胞增高值 + 0.762*CRP增高值，模型R方值为0.823，意味着年龄,性别,术前红细胞压体积%,术后红细胞压体积%,术前血容量（L）,白细胞增高值,CRP增高值可以解释隐性失血（ml）的82.3%变化原因。对模型进行F检验时发现模型通过F检验(F=17.243，p=0.000<0.05)，也即说明年龄,性别,术前红细胞压体积%,术后红细胞压体积%,术前血容量（L）,白细胞增高值,CRP增高值中至少一项会对隐性失血（ml）产生影响关系，另外，针对模型的多重共线性进行检验发现，模型中有VIF值大于5，但是小于10，意味着可能存在着一定的共线性问题，可使用岭回归或者逐步回归解决共线性问题；同时也建议检查相关关系紧密的自变量，剔除掉相关关系紧密的自变量后，重新进行分析。最终具体分析可知：

年龄的回归系数值为-1.033(t=-0.520，p=0.607>0.05)，意味着年龄并不会对隐性失血（ml）产生影响关系。

性别的回归系数值为-94.909(t=-0.884，p=0.385>0.05)，意味着性别并不会对隐性失血（ml）产生影响关系。

术前红细胞压体积%的回归系数值为126.579(t=9.944，p=0.000<0.01)，意味着术前红细胞压体积%会对隐性失血（ml）产生显著的正向影响关系。

术后红细胞压体积%的回归系数值为-116.322(t=-9.545，p=0.000<0.01)，意味着术后红细胞压体积%会对隐性失血（ml）产生显著的负向影响关系。

术前血容量（L）的回归系数值为131.857(t=3.118，p=0.004<0.01)，意味着术前血容量（L）会对隐性失血（ml）产生显著的正向影响关系。

白细胞增高值的回归系数值为-0.131(t=-0.005，p=0.996>0.05)，意味着白细胞增高值并不会对隐性失血（ml）产生影响关系。

CRP增高值的回归系数值为0.762(t=0.402，p=0.691>0.05)，意味着CRP增高值并不会对隐性失血（ml）产生影响关系。

总结分析可知：术前红细胞压体积%, 术前血容量（L）会对隐性失血（ml）产生显著的正向影响关系。以及术后红细胞压体积%会对隐性失血（ml）产生显著的负向影响关系。但是年龄, 性别, 白细胞增高值, CRP增高值并不会对隐性失血（ml）产生影响关系。

模型预测

隐性失血（ml）	=	-1029.8321000761
年龄	=	输入数字
性别	=	输入数字
术前红细胞压体积%	=	输入数字
术后红细胞压体积%	=	输入数字
术前血容量（L）	=	输入数字
白细胞增高值	=	输入数字
CRP增高值	=	输入数字

线性回归分析结果-简化格式

	回归系数	95% CI	共线性诊断	
			VIF	容忍度
常数	-1029.832<br>(-2.007)	-2084.566 ~ 24.902	-	-
年龄	-1.033<br>(-0.520)	-5.113 ~ 3.048	1.335	0.749
性别	-94.909<br>(-0.884)	-315.566 ~ 125.748	3.106	0.322
术前红细胞压体积%	126.579**<br>(9.944)	100.413 ~ 152.745	5.211	0.192
术后红细胞压体积%	-116.322**<br>(-9.545)	-141.372 ~ -91.271	4.899	0.204
术前血容量（L）	131.857**<br>(3.118)	44.937 ~ 218.776	2.444	0.409
白细胞增高值	-0.131<br>(-0.005)	-50.116 ~ 49.855	1.122	0.892
CRP增高值	0.762<br>(0.402)	-3.132 ~ 4.656	1.117	0.895
样本量	34			
R^2	0.823			
调整R^2	0.775			
F值	$F(7,26)=17.243, p=0.000$			

备注：因变量 = 隐性失血（ml）

D-W值 = 2.439

* $p<0.05$ ** $p<0.01$ 括号里面为t值

模型结果图

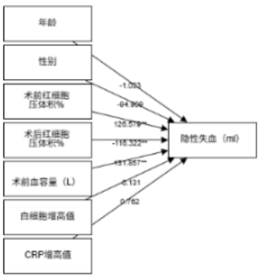

下载　展示数值

模型汇总(中间过程)

R	R^2	调整R^2	模型误差RMSE	DW值	AIC值	BIC值
0.907	0.823	0.775	148.431	2.439	452.496	464.707

分析建议

回归分析用于研究X(定量或定类)对Y(定量)的影响关系，是否有影响关系，影响方向及影响程度情况如何；

第一：模型*R*方值分析模型拟合情况，比如0.5则表示模型有50%的拟合程度；

第二：调整*R*方值用于惩罚任意放置过多的X，通常情况下使用较少。

智能分析

从上表可知，将年龄,性别,术前红细胞压体积%,术后红细胞压体积%,术前血容量（L）,白细胞增高值,CRP增高值作为自变量，而将隐性失血（ml）作为因变量进行线性回归分析，从上表可以看出，模型*R*方值为0.823，意味着年龄,性别,术前红细胞压体积%,术后红细胞压体积%,术前血容量（L）,白细胞增高值,CRP增高值可以解释隐性失血（ml）的82.3%变化原因。

ANOVA表格(中间过程)

	平方和	df	均方	F	p 值
回归	3477508.831	7	496786.976	17.243	0.000
残差	749082.278	26	28810.857		
总计	4226591.109	33			

分析建议

*F*检验用于检验回归模型是否有意义；

第一：如果模型通过*F*检验($p<0.05$)，说明模型有意义，至少有一个X会对Y产生影响；

第二：如果模型没有通过*F*检验($p>0.05$)，说明模型构建无意义，X均不会对Y产生影响。

智能分析

从上表可知，对模型进行*F*检验时发现模型通过*F*检验($F=17.243$，$p=0.000<0.05$)，也即说明模型构建有意义。

回归系数(中间过程) (n=34)

	非标准化系数		标准化系数	t	p	95% CI	VIF
	B	标准误	Beta				
常数	-1029.832	513.120	-	-2.007	0.055	-2084.566 ~ 24.902	-
年龄	-1.033	1.985	-0.050	-0.520	0.607	-5.113 ~ 3.048	1.335
性别	-94.909	107.348	-0.129	-0.884	0.385	-315.566 ~ 125.748	3.106
术前红细胞压体积%	126.579	12.729	1.874	9.944	0.000**	100.413 ~ 152.745	5.211
术后红细胞压体积%	-116.322	12.187	-1.744	-9.545	0.000**	-141.372 ~ -91.271	4.899
术前血容量（L）	131.857	42.286	0.402	3.118	0.004**	44.937 ~ 218.776	2.444
白细胞增高值	-0.131	24.317	-0.000	-0.005	0.996	-50.116 ~ 49.855	1.122
CRP增高值	0.762	1.894	0.035	0.402	0.691	-3.132 ~ 4.656	1.117

备注：因变量 = 隐性失血（ml）

* $p<0.05$ ** $p<0.01$

coefPlot

回归系数95% CI

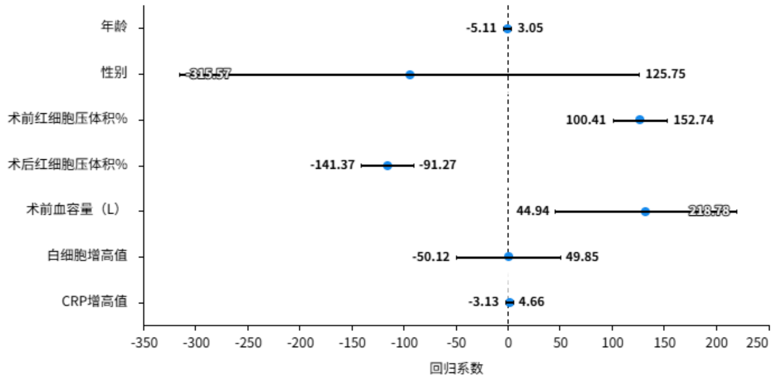

复　下

制 载 尺寸： 样式

样本缺失情况汇总

项	样本数	占比
有效样本	34	100.0%
排除无效样本	0	0.0%
总计	34	100%

分析建议

上表格展示真实进入算法模型时有效样本和排除在外的无效样本情况等。

第一：上表格中'有效样本'指所有分析项均有数据的样本总数，'排除无效样本'指任意一个分析项出现缺失的样本总数；

第二：如果某样本在任意一个分析项上出现缺失数据（即排除无效样本），该类样本无法进入模型分析，模型只能针对有效样本进行分析；

第三：可通过'通用方法'里面的描述分析检查各分析项样本情况，也可在右上角'查看数据'查看具体数据。

参考文献

【1】The SPSSAU project (2025). SPSSAU. (Version 25.0) [Online Application Software]. Retrieved from https://www.spssau.com.

【2】周俊,马世澎. SPSSAU科研数据分析方法与应用.第1版[M]. 电子工业出版社,2024.

【3】Sun Dao-de. Selection of the Linear Regression Model According to the Parameter Estimation[J]. Wuhan University Journal of Natural Sciences, 2000, 5(4):400-405.

【4】Barassi M R. Microeconometrics; Methods and Applications by A. Colin Cameron; Pravin K. Trivedi[J]. 2005.

【5】威廉H·格林.经济计量分析[M].北京:中国社会科学出版社,1998.

【6】张厚粲, 徐建平. 现代心理与教育统计学.第3版[M]. 北京师范大学出版社, 2009.
